# 

**Published:** 1854-09

**Authors:** 


*Return of Prof. Flint.*—Our senior sailed from Liverpool on the 23d of August, and we shall probably greet his return before this number reaches our readers. His European tour, or rather Parisian residence, has been both profitable and pleasant to him, and the effect of a few months of change of scene and occupation on our industrious colleague, must needs be beneficial.

